# Genetic determinants of swimming motility in the squid light-organ symbiont *Vibrio fischeri*

**DOI:** 10.1002/mbo3.96

**Published:** 2013-06-12

**Authors:** Caitlin A Brennan, Mark J Mandel, Mattias C Gyllborg, Krista A Thomasgard, Edward G Ruby

**Affiliations:** Department of Medical Microbiology and Immunology, University of WisconsinMadison, Wisconsin

**Keywords:** Chemotaxis, *Euprymna scolopes*, Flagellar motility, symbiosis

## Abstract

Bacterial flagellar motility is a complex cellular behavior required for the colonization of the light-emitting organ of the Hawaiian bobtail squid, *Euprymna scolopes*, by the beneficial bioluminescent symbiont *Vibrio fischeri*. We characterized the basis of this behavior by performing (i) a forward genetic screen to identify mutants defective in soft-agar motility, as well as (ii) a transcriptional analysis to determine the genes that are expressed downstream of the flagellar master regulator FlrA. Mutants with severe defects in soft-agar motility were identified due to insertions in genes with putative roles in flagellar motility and in genes that were unexpected, including those predicted to encode hypothetical proteins and cell division–related proteins. Analysis of mutants for their ability to enter into a productive symbiosis indicated that flagellar motility mutants are deficient, while chemotaxis mutants are able to colonize a subset of juvenile squid to light-producing levels. Thirty-three genes required for normal motility in soft agar were also downregulated in the absence of FlrA, suggesting they belong to the flagellar regulon of *V. fischeri*. Mutagenesis of putative paralogs of the flagellar motility genes *motA motB,* and *fliL* revealed that *motA1 motB1*, and both *fliL1* and *fliL2*, but not *motA2* and *motB2*, likely contribute to soft-agar motility. Using these complementary approaches, we have characterized the genetic basis of flagellar motility in *V. fischeri* and furthered our understanding of the roles of flagellar motility and chemotaxis in colonization of the juvenile squid, including identifying 11 novel mutants unable to enter into a productive light-organ symbiosis.

## Introduction

Flagellar motility is an environmentally regulated behavior by which a bacterium propels itself through its surroundings, directed by behavior-modifying machinery such as the chemotaxis system (Adler 1966; Henrichsen 1972; reviewed in Macnab 1996; and McCarter 2006). Within the unique environments present in different host–microbe associations, both flagellar motility and the flagellum itself can play important roles in bacterial transit, niche specificity, effector secretion, biofilm formation, host recognition, and gene regulation (Young et al. 1999; Hayashi et al. 2001; Butler and Camilli 2004; Lemon et al. 2007; Liu et al. 2008). While the process of flagellar motility is difficult to study in most host–microbe interactions, the symbiosis between the bioluminescent, gram-negative bacterium *Vibrio fischeri* and its host the Hawaiian bobtail squid, *Euprymna scolopes,* is an ideal model in which to study how this critical behavior mediates symbiotic initiation. The squid–vibrio mutualism provides a vast array of tools to study the roles of the flagellum and flagellar motility both in culture and throughout the development and persistence of the symbiosis (Nyholm et al. 2000; DeLoney-Marino et al. 2003; Mandel et al. 2012; Brennan et al. 2013).

The association between *V. fischeri* and *E. scolopes* begins as soon as the juvenile squid hatches in seawater containing the symbiont. Colonization is a sequential process in which the bacteria initiate the association, accommodate to the light-organ environment, and persist in epithelium-lined crypts for the lifetime of the squid (Nyholm and McFall-Ngai 2004). The steps mediating symbiotic initiation involve a complex exchange of signals and responses between *V. fischeri* and the juvenile squid (Nyholm and McFall-Ngai 2004; Visick and Ruby 2006; Mandel et al. 2012). To enter into the symbiosis, *V. fischeri* cells must migrate from external aggregates, through mucus to the pores of the light organ, and finally into crypts deep within the tissue. Remarkably, essentially all other species of bacteria are excluded from completing this path. Flagellar biosynthesis by *V. fischeri* and its subsequent use for motility are essential for the initiation process (Graf et al. 1994; Millikan and Ruby 2003). While the role of chemotaxis in initiation is less well characterized, a mutant disrupted in the chemotaxis response regulator CheY is defective in competition with wild-type *V. fischeri* (Hussa et al. 2007). Other studies have shown that alterations to motility – both increasing and decreasing motility rates – result in defects in the initiation kinetics (Millikan and Ruby 2002, 2004).

*Vibrio fischeri* is motile by means of a unipolar tuft of ∼2–7 sheathed flagella, rather than by the peritrichous flagella of the model organisms *Escherichia coli* and *Salmonella enterica* serovar Typhimurium (Allen and Baumann 1971; Macnab 2003). The use of multiple polar flagella in *V. fischeri* is unique even among the well-studied *Vibrio* species (McCarter 2006): *Vibrio cholerae* bears a single polar flagellum and *V. parahaemolyticus* presents either a single sheathed polar flagellum or multiple unsheathed lateral flagella, depending on its environment (Shinoda and Okamoto 1977; Freter et al. 1981; McCarter and Silverman 1990). These structural differences suggest that flagellar biosynthesis is uniquely adapted in this genus. Within Gram-negative bacteria, the complex regulation of genes involved in flagellar motility occurs through a multiple-tiered cascade of events. In *E. coli*, early flagellar genes are activated in a σ^70^-dependent manner by the master regulator FlhDC. Late flagellar genes are then activated under the control of FliA (σ^28^) (Iino et al. 1988; Arnosti and Chamberlin 1989). In contrast, the regulation of flagellar genes by *V. cholerae* occurs in a four-tiered regulatory cascade controlled by the σ^54^-dependent activator, FlrA, which activates the early flagellar genes, such as those involved in the MS ring structure (Klose and Mekalanos 1998b). Late flagellar genes, including those encoding hook and motor proteins, are expressed in a sequential manner by either the two-component regulator FlrC or the FliA sigma factor (Prouty et al. 2001). In *V. fischeri*, amotile cells are observed in strains encoding single mutations in the genes *rpoN* (which encodes σ^54^), *flrA,* or *flrC*, suggesting that flagellar regulation occurs through a regulatory cascade that is similar to *V. cholerae* (Millikan and Ruby 2003; Wolfe et al. 2004; Hussa et al. 2007). Flagellar activators do not exclusively regulate flagellar gene products; instead, they control both virulence and metabolic signatures in other bacteria (Pruss et al. 2003; Kapatral et al. 2004; Syed et al. 2009) and have been similarly implicated in the modulation of unknown symbiotic factors in *V. fischeri* (Millikan and Ruby 2003).

Genome scanning predicts that the genetic basis of flagellar motility and chemotaxis is complex in *V. fischeri*, as this organism's two chromosomes encode paralogs of multiple *E. coli* flagellar genes as well as 43 predicted methyl-accepting chemotaxis proteins (MCPs) (Ruby et al. 2005; McCarter 2006; Mandel et al. 2008). Similarly large numbers of MCPs have been observed in other sequenced microbes not in the *Enterobacteriaceae*, but their function(s) remains poorly described (Miller et al. 2009). Several genetic studies have identified the genes involved in proper flagellar elaboration in bacteria with polar flagellar systems (Kim and McCarter 2000; Overhage et al. 2007). In these bacteria, additional proteins are important for motility, including the regulators FlhF and FlhG, which control flagellar number in *V. cholerae* (Correa et al. 2005). The continuing discovery of new polar flagellum-specific genes (Sommerlad and Hendrixson 2007; Cameron et al. 2008; Morris et al. 2008; Moisi et al. 2009), as well as differences in flagellar structure, suggest there exist additional novel structural components and/or regulatory factors that are critical for flagellar motility of *V. fischeri*.

In the work described here, we sought to understand the genetic basis of flagellar motility, an essential cellular behavior for the host-associated lifestyle of *V. fischeri*. We performed both forward and reverse genetic analyses, coupled with transcriptional profiling, to identify the genes that contribute to normal motility during symbiosis.

## Experimental Procedures

### Bacterial strains and media

Strains and plasmids used in this work are listed in Table S1. *Vibrio fischeri* strains are derived from the squid isolate ES114 (Boettcher and Ruby 1990) and were grown at 28°C in either Luria-Bertani salt (LBS) medium (per L, 10 g Bacto-tryptone, 5 g yeast extract and 20 g NaCl, 50 mL 1 mol/L Tris buffer, pH 7.5, in distilled water) or seawater-based tryptone (SWT) medium (per L, 5 g Bacto-tryptone, 3 g yeast extract, 3 mL glycerol, 700 mL Instant Ocean [Aquarium Systems, Inc, Mentor, OH] at a salinity of 33–35 ppt, and 300 mL distilled water). When used to support overnight growth, SWT was supplemented with 50 mmol/L Tris buffer, pH 7.5. *Escherichia coli* strains, as used for cloning, were grown at 37°C in Luria-Bertani medium or brain heart infusion medium (BD, Sparks, MD). When appropriate, antibiotics were added to media at the following concentrations: erythromycin (erm), 5 μg/mL for *V. fischeri* and 150 μg/mL for *E. coli*; kanamycin (kan), 100 μg/mL for *V. fischeri* and 50 μg/mL for *E. coli*; and chloramphenicol (cam), 2.5 μg/mL for *V. fischeri* and 25 μg/mL for *E. coli*. Growth media were solidified with 1.5% agar as needed.

### Construction and motility screening of an arrayed transposon mutant collection

To investigate the genetic basis of flagellar motility, we conducted random mutagenesis with pMJM10, a conjugatable plasmid that encodes an erythromycin-resistant transposon. Plasmid pMJM10 is a derivative of pEVS170 (Lyell et al. 2008) that includes outward-facing T7 promoters on the transposon and MseI/Tsp509I sites in the vector backbone outside of the transposon. The tranpsoson was built as an optimized vector to conduct TraSH analysis (Sassetti et al. 2001) in *V. fischeri* and it performs comparable to pEVS170 for traditional forward genetic analysis. The plasmid backbone encodes a Tn*5* transposase, kanamycin resistance, and an R6Kγ (pi-dependent) origin of replication that does not replicate in *V. fischeri*. In 10–15% of instances the plasmid backbone is retained in *V. fischeri* illegitimately (Lyell et al. 2008); these isolates are identified by their kanamycin resistance and removed from the study.

Construction from pEVS170 was accomplished as follows. First, oligonucleotides T7US-F2 and T7US-R2 were 5'-phosphorylated with T4 polynucleotide kinase (Promega, Madison, WI) and then annealed. The resulting heteroduplex was introduced into the SpeI and Bme1580I sites of pEVS170. The resulting plasmid was named pMJM8 (contains a single outward-facing T7 promoter at the upstream end of the transposon, relative to the orientation of the *erm* cassette). Second, vector sequence adjacent to the transposon upstream end was modified to introduce MseI and Tsp509I sites by site-directed mutagenesis. The QuikChange II Kit (Stratagene, La Jolla, CA) was used according to the manufacturer's instructions, and mutation of the hexanucleotide GGGGGG to TTAATT with oligonucleotides MseTspUS-F and MseTspUS-R introduced the new restriction endonuclease cleavage sites for the resulting plasmid pMJM9. Finally, the downstream T7 promoter and MseI/Tsp509I sites were introduced in a single step. pMJM9 served the template for a polymerase chain reaction (PCR) in which T7DS-pcrF2 and T7DS-pcrR amplified most of the plasmid, introducing the changes. The resulting PCR product was digested with KpnI (sites were included in the primers) and DpnI (to digest the template DNA), then self-ligated to generate pMJM10. The transposon sequence and its immediate flanking DNA were confirmed by DNA sequencing at the University of Wisconsin Biotechnology Center.

The construction of the MB mutant collection is illustrated in detail in Figure S1. Briefly, we mutagenized *V. fischeri* ES114 by Tn*erm* transposition and arrayed mutants into 96-well trays for individual analysis. We screened the arrayed mutants for strains that contained a transposon insertion (erythromycin resistant), but that additionally did not retain the donor plasmid (kanamycin sensitive). These strains were rearrayed, frozen as glycerol stocks, and saved for further analysis.

For soft-agar motility screening, strains were inoculated from the rearrayed cultures into 100 μL SWT buffered with 50 mmol/L Tris and grown overnight. Omnitrays (Nunc, Rochester, NY) containing SWT 0.3% agar were inoculated, in duplicate, with 1 μL of each overnight culture using a 96-pin replicator (V&P Scientific, San Diego, CA). Soft-agar plates were then incubated at 28°C for 4–6 h and examined for alterations in swim colony morphology.

The final library contains 23,904 mutant strains and is termed the MB Collection. This collection contains only trays that, upon screening for motility phenotypes, provided reproducible phenotypes across the entire agar tray during the motility screen. We observed a correlation between aberrant motility screen results and subsequent inability to regrow strains from plate freezer stocks. We attributed such results to harsh growth conditions during passage of the strains (e.g., inadvertent heat shock). In this manner, motility screening provided a quality control step for the entire collection. Trays that did not pass this step were not included in the final collection or in the screen results.

The insertion site of the candidate amotile mutants were identified using arbitrarily primed PCR as previously described (Caetano-Anolles 1993; O'Toole et al. 1999). Briefly, the transposon junction site was amplified from a diluted overnight culture in two successive rounds of PCR using primer sets ARB1/170Ext3, followed by ARB2/170Int3 (Table S2). The sample was submitted for sequencing to the DNA Sequencing Center at the University of Wisconsin Biotech Center (Madison, WI) with primer 170Seq1. In the event that this method did not yield a high quality sequence, arbitrarily primed PCR was repeated on purified genomic DNA collected using the Wizard SV Genomic DNA Purification kit (Promega, Madison, WI).

### Motility and growth studies

For individual soft-agar motility assays, cells were grown to an OD_600_ of ∼0.3–0.4. Cultures were then normalized to an OD_600_ of 0.3, and 2 μL of each strain were inoculated into plates containing SWT supplemented with 0.3% agar. Plates were grown at 28°C for ∼10–12 h, at which point the diameter of each swim colony was measured and plates were photographed, if desired.

In preparation for all microscopy, cells were grown in SWT broth with shaking at 28°C to an OD_600_ of ∼0.3. For liquid motility and cell morphology studies, live cells were applied to a slide and examined by phase-contrast microscopy under a 40× objective. For examination of flagellar structures, cells were applied to Pioloform-coated copper grids (Ted Pella Co., Tustin, CA) for 5 min, washed with sterile water for 30 sec, and negatively stained for 1 min with NanoW (Nanoprobes, Yaphank, NY). Grids were immediately examined using a Philips CM120 transmission electron microscope (University of Wisconsin Medical School Electron Microscope Facility, Madison, WI).

For growth curves, overnight cultures were used to inoculate 100 μL SWT, arrayed in a 96-well plate, and a plate reader was used to measure OD_600_ over 8 h of growth at 28°C with shaking. Data represent the mean and standard error of the mean for three biological replicates.

### Molecular cloning

Campbell-type (insertion-duplication) mutagenesis using the suicide vector pEVS122 (Dunn et al. 2005) was performed to generate disruptions in *motA1 motB1 motA2 motB2 fliL1 and fliL2*. Using the following primer pairs, ∼200 bp of homology near the 5' end of each open reading frame (ORF) was amplified for each gene by PCR: *motA1*, motA1_campbellF, and motA1_campbellR; *motB1*, motB1_campbellF, and motB1_campbellR; *motA2*, motA2_campbellF, and motA2_campbellR; *motB2*, motB2_campbellF, and motB2_campbellR; *fliL1*, fliL1_campbellF, and fliL1_campbellR; and *fliL2*, fliL2_campbellF, and fliL2_campbellR (Table S2). The primer pairs also added XmaI and SphI restriction enzyme sites, which were used to clone the amplified products into the XmaI/SphI-digested pEVS122 using standard techniques. The resulting constructs were conjugated into *V. fischeri* ES114 (MJM1100 isolate) as previously described (Stabb and Ruby 2002).

Complementation constructs were generated using the pES213-derived pVSV105 (Dunn et al. 2006). For *flgOP flgT VF_1491 mutS*, and *fliL2*, each ORF and 350 bp upstream was amplified by PCR using the following primer pairs: *flgOP*, flgO_compF, and flgP_compR; *flgT*, flgT_compF, and flgT_compR; *VF_1491*, 1491_compF, and 1491_compR; *mutS*, mutS_compF, and mutS_compR; and *fliL2*, fliL2_compF, and fliL2_compR. For *flgP* (primer pair flgP_compF and flgP_compR), *amiB* (primer pair amiB_compF and amiB_compR), and *mukB* (primer pair mukB_compF and mukB_compR), which are located within predicted operons, amplification introduced a ribosome-binding site, such that gene expression is driven by the *lacZα* promoter. The amplified products were directionally cloned into the multiple cloning site of pVSV105 (Dunn et al. 2006), using standard molecular techniques. Both the complementation construct and vector control were conjugated into wild-type *V. fischeri* and the relevant mutant by tri-parental mating (Stabb and Ruby 2002).

LacZ transcriptional fusions were constructed by amplification of the promoter region and subsequent cloning into SalI/AvrII-digested pAKD701, upstream of a promoterless *lacZ* (Dunn and Stabb 2008). The primer pairs were designed to amplify ∼350 bp upstream of the start codon and the first 120 bp of the ORF, as well as introduce SalI and AvrII restriction enzyme sites into the product. Primer pairs are as follows: *fliEp*, fliE_fusionF, and fliE_fusionR; *flhAp*, flhA_fusionF, and flhA_fusionR; *flrBp*, flrB_fusionF, and flrB_fusionR; *fliKp*, fliK_fusionF, and fliK_fusionR; *fliKp*, fliK_fusionF, and fliK_fusionR; *flgAp*, flgA_fusionF, and flgA_fusionR; *flgBp*, flgB_fusionF, and flgB_fusionR; *flaDp*, flaD_fusionF, and flaD_fusionR; *motXp*, motX_fusionF, and motX_fusionR; *motA1p*, motA1_fusionF, and motA1_fusionR; *flgOp*, flgO_fusionF, and flgO_fusionR; and *flgTp*, flgT_fusionF, and flgT_fusionR. Reporter constructs were introduced to wild-type *V. fischeri* and the *flrA*::Tn*erm* mutant by tri-parental mating.

### Transcriptional studies

For microarray analysis, wild-type and Δ*flrA* cells were grown in SWT with shaking at 28°C with an OD_600_ of ∼0.5. RNA from two biological replicates was harvested, labeled, and hybridized to the *V. fischeri* Affymetrix chip, as previously described (Wang et al. 2010; Wier et al. 2010). Data were deposited into the Gene Expression Omnibus (accession # GSE45772). In β-galactosidase assay studies, wild type or the *flrA*::Tn*erm* mutant harboring reporter constructs were similarly cultured in SWT medium with shaking at 28°C to an OD_600_ of ∼0.5. β-galactosidase activity was measured from four biological replicates, using a modified microtiter dish method (Slauch and Silhavy 1991; Studer et al. 2008). The relative units of β-galactosidase activity were calculated using the following formula: (*V*_max_)/(OD_600_) × volume (mL).

### Bioinformatic analyses

Microarray analysis was performed with Cyber-T software (Baldi and Long 2001), using a cut-off of at least a twofold change and a *P*-value <0.01. The Database of prOkaryotic OpeRons (DOOR) was used to analysis of the *V. fischeri* ES114 genome and predict operon structure (Dam et al. 2007). Functional classes were informed, in part, by the cellular role categorization provided by the J. Craig Venter Institute Comprehensive Microbial Resource (Peterson et al. 2001).

### Squid experiments

Newly hatched squid (<6 h posthatch) were colonized using standard methods (Naughton and Mandel 2012), except that the final culturing of the *V. fischeri* inoculum was in SWT broth. Briefly, squid were transiently exposed to ∼5000 colony-forming-units (CFU) per mL of a given strain in filter-sterilized Instant Ocean (FSIO), set at 33–35 ppt, for the inoculation time listed in a particular experiment. At the end of the inoculation period, squid were transferred to uninoculated FSIO, and the luminescence of individual squid was measured using a TD-20/20 luminometer at the conclusion of the experiment (Turner Biosystems, Sunnyvale, CA). Squid were considered colonized to luminous levels if the luminometer measurement was above 10 relative light units (RLUs); background readings were usually <2 RLUs. Squid colonized with wild-type *V. fischeri* harbored, on average, ∼300,000 CFU/squid, whereas nonluminous squid (<10 RLU) generally carried fewer than <1000 CFU/squid (data not shown). Batches of 9–10 squid were used for each condition, and each experiment was performed at least in triplicate.

## Results

### Construction and soft-agar motility screening of a *V. fischeri* transposon mutant collection

To investigate the genetic basis of flagellar motility, we randomly mutagenized *V. fischeri* ES114 by Tn*5* mutagenesis with the erythromycin resistance-encoding transposon carried on pMJM10, and arrayed the mutants into 96-well trays for individual analysis. Because a subset of transposon insertions also retains the transposon/transposase donor plasmid (Lyell et al. 2008), we screened for strains that contained a transposon insertion (i.e., erythromycin resistant), but that did not retain the donor plasmid (i.e., kanamycin sensitive). The resulting 23,904 strains, named the MB mutant collection, were rearrayed, frozen as glycerol stocks, and used for further analysis (Fig. S1 and Experimental Procedures)

**Figure 1:**
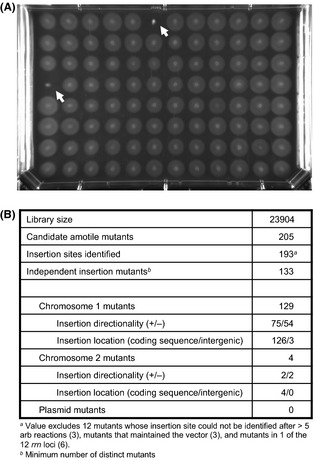
Soft-agar motility screening of a *Vibrio fischeri* transposon mutant library. (A) A representative soft-agar motility plate. White arrows indicate strains considered as candidate amotile mutants. (B) Summary of the characteristics of the transposon mutant library and the results of the soft-agar motility screen. Directionality refers to the direction of the transposon's *erm* cassette relative to chromosome nucleotide orientation as deposited in GenBank.

*Vibrio fischeri* mutants that were defective in swimming motility were identified by performing a multiplex soft-agar motility assay in agar trays. Briefly, each tray of mutant strains was replicated to fresh medium and grown overnight. The cultures were then pin replicated into trays containing SWT medium containing 0.3% agar in which the cells from all 96-wells swam out from their center point in synchrony (Fig. 1A). Using this assay, 205 mutants characterized as candidate amotile strains were selected; almost all (97%) of these transposon insertion sites were located on Chromosome 1 (Fig. 1B). One representative mutant was selected per gene (Table S1) and analyzed in a controlled soft-agar motility assay in which culture densities were normalized by optical density. Mutants with <30% of a wild-type migration distance were designated as severely affected in flagellar motility (Table 1). Of the 66 representative mutants, 21 had motility that was not as severely affected as the 30% threshold, yet still exhibited motility reproducibly less than wild type in the individual assays (Table 2). These reduced-motility mutants largely mapped to the lipopolysaccharide biosynthesis locus, defects in which have been tied to altered motility in several organisms (Girgis et al. 2007; Cullen and Trent 2010). However, three mutants in this category had insertions in homologs of flagellar genes. These genes include *flgL*, encoding a hook-associated protein, as well as *flaA* and *flaD*, two of the six flagellin-encoding genes in the *V. fischeri* genome. The phenotype of the *flaA* mutant is consistent with a previous report (Millikan and Ruby 2004), but the requirement for *flaD* in normal soft-agar motility represents a novel finding. Other mutants within this category were disrupted in genes whose mutation is likely to cause growth defects, or in genes encoding hypothetical proteins. As these 21 moderately defective mutants exhibited a weaker phenotype upon secondary screening, they were not pursued in this study.

**Table 1:** Characterization of transposon mutants with greatly reduced (<30% of normal) soft-agar motility

Function	ORF	Gene	Description	% wild-type motility^1^	FlrA activation^2^	# independent mutants	Predicted operon structure^3^
Regulation	VF_0387	*rpoN*	RNA polymerase sigma-54 factor	0	NS^4^	7	*kdsDC-0390-lptAB-**rpoN**-hpf-ptsN-yhbJ-0383*^5^
	VF_1856	*flrA*	Sigma-54-dependent regulator	0	ND	2	***flrA***
	VF_1854	*flrC*	Two-component response regulator	0	8.1	4	***flr**B**C***
	VF_1855	*flrB*	Two-component sensor kinase	17	16	1	***flrB**C*
	VF_1834	*fliA*	RNA polymerase sigma-28 factor	0	NS	2	f*lhAFG-**fliA-**cheYZAB-1829-1828-cheW-1825*
	VF_1835	*flhG*	Flagellar synthesis regulator	0	NS	1	***flh**AF**G**-fliA-cheYZAB-1829-1828-cheW-1825*
	VF_1836	*flhF*	Flagellar regulator	23	2.6	4	***flh**A**F**G-fliA-cheYZAB-1829-1828-cheW-1825*
Structure/Secretion	VF_1837	*flhA*	Flagellar biosynthesis protein	0	NS	5	***flhA**FG-fliA-cheYZAB-1829-1828-cheW-1825*
	VF_1840	*fliR*	Flagellar biosynthesis protein	0	2.9	1	***fli**L1MNOPQ**R***
	VF_1844	*fliN*	Flagellar motor switch component	0	6.4	1	***fli**L1M**N**OPQR*
	VF_1845	*fliM*	Flagellar motor switch protein	0	6.8	1	***fli**L1**M**NOPQR*
	VF_1846	*fliL1*	Flagellar basal body–associated protein	0	8.1	1	***fliL1**MNOPQR*
	VF_1847	*fliK*	Flagellar hook length control protein	0	9.2	2	***fliK***
	VF_1849	*fliI*	Flagellum-specific ATP synthase	0	NS	2	***fli**H**I**J*
	VF_1850	*fliH*	Flagellar assembly protein	0	NS	3	***fliH**IJ*
	VF_1851	*fliG*	Flagellar motor switch protein	0	6.8	5	***fli**EF**G***
	VF_1852	*fliF*	Flagellar M ring protein	0	6.6	2	***fli**E**F**G*
	VF_1860	*fliD*	Flagellar hook-associated protein 2	19	7.4	3	*flaG-**fliD** -flaI-fliS*
	VF_1868	*flgK*	Flagellar hook-associated protein 1	0	41	4	***flgK**L*
	VF_1870	*flgI*	Flagellar P ring protein	0	3.5	5	***flg**FGH**I***
	VF_1871	*flgH*	Flagellar L ring protein	0	6.3	1	***flg**FG**H**I*
	VF_1872	*flgG*	Flagellar distal rod protein	0	12	2	***flg**F**G**HI*
	VF_1873	*flgF*	Flagellar proximal rod protein	0	9.6	2	***flgF**GHI*
	VF_1874	*flgE*	Flagellar hook protein	0	5.7	1	***flgE***
	igVF_1874		(intergenic region)	0	ND	1	
	VF_1875	*flgD*	Flagellar hook capping protein	0	7.5	2	***flg**BC**D***
	VF_1882	*flgN*	Flagellar chaperone	0	2.5	1	***flg**M**N***
Motor	VF_0714	*motA1*	Flagellar motor protein	0	6.3	1	***motA1**B1*
	VF_0715	*motB1*	Flagellar motor protein	0	3.4	1	***mot**A1**B1***
	VF_0926	*motY*	Flagellar motor protein	0	2.6	2	***motY***
	VF_2317	*motX*	Flagellar motor protein	0	11	2	***motX***
Chemotaxis	VF_1826	*cheW*	Chemotaxis coupling protein	10	2.6	1	*flhAFG-fliA-cheYZAB-1829-1828-**cheW**-1825*
	VF_1830	*cheB*	Chemotaxis methyl esterase	24	2.1	2	*flhAFG-fliA-**che**YZA**B**-1829-1828-cheW-1825*
	VF_1831	*cheA*	Chemotaxis histidine autokinase	7	2.0	6	*flhAFG-fliA-**che**YZ**A**B-1829-1828-cheW-1825*
	VF_1832	*cheZ*	CheY phosphatase	8	2.2	1	*flhAFG-fliA-**che**Y**Z**AB-1829-1828-cheW-1825*
	VF_1833	*cheY*	Chemotaxis response regulator	6	2.2	1	*flhAFG-fliA-**cheY**ZAB-1829-1828-cheW-1825*
Unexpected	igVF_0135		(intergenic region)	21	ND	1	
	VF_0534	*mutS*^5^	Methyl-directed mismatch repair protein	0	NS	1	***mutS***
	VF_1491		Hypothetical protein	9	NS	3	***1491***
	VF_1883	*flgP*	Flagellar motility-associated protein	0	8.7	4	***flg**O**P***
	VF_1884	*flgO*	Flagellar motility-associated protein	0	10	2	***flgO**P*
	VF_1885	*flgT*	Flagellar motility-associated protein	0	NS	1	***flgT***
	VF_2326	*amiB*	N-acetylmuramoyl-l-alanine amidase II	0	NS	1	*yjeE-**amiB-**mutL-miaA*
	VF_A0430	*mukF*	Calcium-binding protein involved in chromosome partioning	23	NS	1	*smtA-**mukF**EB*
	VF_A0432	*mukB*	Fused chromosome partitioning protein	22	NS	2	*smtA-**muk**FE**B***

ORF, open reading frame; ND, not determined.

1 As scored by the normalized soft-agar motility assay described in Experimental Procedures.

2 FlrA activation is defined as the fold change of gene expression in wild type as compared to Δ*flrA*, as determined by microarray analysis.

3 Predicted operon structure based on DOOR (Database of prOkaryotic OpeRons) analysis of the *Vibrio fischeri* genome. Bold font indicates gene of interest in the predicted operon.

NS, not significant at fold change ≥2 and *P* ≤ 0.01.

5 Four-digit numbers indicate locus tags and should be read as preceded by “*VF_*”.

6 When *mutS* is expressed *in trans*, the complemented strain does not regain the ability to swim through soft agar (data not shown), indicating that *mutS* expression does not directly mediate flagellar motility in this strain. MutS is a protein involved in DNA mismatch repair, and the loss of this function results in strains with higher mutation rates. We hypothesize that the mutagenic nature of the *mutS* strain enabled a secondary mutation that is responsible for the amotile phenotype, and we will not follow up on this mutant in this study.

**Table 2:** Description of mutants with moderately reduced (30–90% of normal) soft-agar motility

ORF	Gene	Description	% wild-type motility^1^	FlrA activation^2^	# independent insertions	Predicted operon structure^3^	Potential reason for defect
VF_0077		2-polyprenyl-3-methyl-5-hydroxy-6-metoxy-1,4-benzoquinol methylase	74	NS^5^	1	***0077-**yihI-0079*^5^	Altered LPS/surface structure
VF_0167	*rffH*	Glucose-1-phosphate thymidylyltransferase	44	NS	1	***rff**G**H-**rfbC-rmlB-rfbX-0171-0172-0173-2581-0174-0175-0176-0177-0178-kpsF*	Altered LPS/surface structure
VF_0169	*rmlB*	dTDP-glucose-4,6-dehydratase	56	NS	1	*rffGH-rfbC-**rmlB-**rfbX-0171-0172-0173-2581-0174-0175-0176-0177-0178-kpsF*	Altered LPS/surface structure
VF_0170	*rfbX*	Polisoprenol-linked O-antigentransporter	49	NS	3	*rffGH-rfbC-rmlB-**rfbX**-0171-0172-0173-2581-0174-0175-0176-0177-0178-kpsF*	Altered LPS/surface structure
VF_0171		Hypothetical protein	40	NS	1	*rffGH-rfbC-rmlB-rfbX-**0171**-0172-0173-2581-0174-0175-0176-0177-0178-kpsF*	Altered LPS/surface structure
VF_0172		O-acetyltransferase	39	NS	1	*rffGH-rfbC-rmlB-rfbX-0171-**0172**-0173-2581-0174-0175-0176-0177-0178-kpsF*	Altered LPS/surface structure
VF_0173		Hypothetical protein	43	NS	1	*rffGH-rfbC-rmlB-rfbX-0171-0172-**0173**-2581-0174-0175-0176-0177-0178-kpsF*	Altered LPS/surface structure
VF_2581		Hypothetical membrane protein	55	NS	2	*rffGH-rfbC-rmlB-rfbX-0171-0172-0173-**2581**-0174-0175-0176-0177-0178-kpsF*	Altered LPS/surface structure
VF_0174		beta-D-GlcNAc beta-1,3-galactosyltransferase	49	NS	3	*rffGH-rfbC-rmlB-rfbX-0171-0172-0173-2581-**0174**-0175-0176-0177-0178-kpsF*	Altered LPS/surface structure
VF_0189		Hypothetical membrane protein	73	NS	1	*0187-0188-**0189**-0190*	Altered LPS/surface structure
VF_0192	*fnlB*	UDP-2-acetamido-2,6-dideoxy-beta-L-talose 4-dehydrogenase	69	NS	2	***fnl**A**B-**rffE-wbjE-0195*-*0196-0197*	Altered LPS/surface structure
VF_0365	*mshB*	Mannose-sensitive hemagglutinin pilin protein	87	NS	1	***mshB***	Unknown
VF_1697		Hypothetical protein	32	NS	4	***1697***	Unknown
igVF_1837		(intergenic region)	30	ND	1		Incomplete flagellar structures
VF_1863	*flaD*	Flagellin	60	52	2	***flaD***	Incomplete flagellar structures
VF_1866	*flaA*	Flagellin	38	32	2	***flaA***	Incomplete flagellar structures
VF_1867	*flgL*	Flagellar hook-associated protein 3	56	8.7	5	***flg**K**L***	Incomplete flagellar structures
VF_2174	*can*	Carbonic anhydrase	80	NS	1	***can***	Growth defect
VF_A0058		Hypothetical protein	51	NS	1	*A0059-**A0058**-A0057*	Unknown
		16S rRNA	56	ND	3		Growth defect
		23S rRNA	56	ND	3		Growth defect

As the genome of *V. fischeri* ES114 encodes 12 *rrn* operons with a high level of identity (Ruby et al. 2005), the specific *rrn* operon(s) disrupted in these mutants cannot be unambiguously identified.

ORF, open reading frame; ND, not determined.

1 As scored by the normalized soft-agar motility assay described in Experimental Procedures.

2 FlrA activation is defined as the fold change of gene expression in wild type as compared to Δ*flrA*, as determined by microarray analysis.

3 Predicted operon structure based on DOOR analysis of the *Vibrio fischeri* genome. Bold font indicates gene of interest in the predicted operon.

4 NS, not significant at fold change ≥2 and *P* ≤ 0.01.

5 Four-digit numbers indicate locus tags and should be read as preceded by “*VF_*”.

Of the 45 representative mutants with severe defects in flagellar motility, the proteins encoded by the disrupted genes were categorized into five functional groups (Table 1): (i) predicted and known flagellar regulatory proteins, such as the flagellar master activator FlrA; (ii) proteins with homologs that are involved in the flagellar secretory/export apparatus or the flagellum structure itself; (iii) motor proteins; (iv) chemotaxis-related proteins; and (v) a final collection of nine mutants that were disrupted in, or upstream of, genes we did not a priori predict to be involved in flagellar motility. Mutants in this fifth group map to two subsets: genes related to cell division and DNA repair, and genes encoding proteins that were of unknown function. There is also one insertion in an intergenic region, whose effect is not clear. In short, this study has identified mutants disrupted in 38 homologs of previously identified motility genes (33 of which are new for *V. fischeri*), and seven additional genes that are noncanonical “motility” genes, but that have severe motility defects when disrupted by a transposon. We next utilized this array of mutants to examine the role of motility in symbiotic initiation.

### Flagellar motility, but not chemotaxis, is required for entrance into a productive symbiosis with juvenile *E. scolopes*

**Figure 2:**
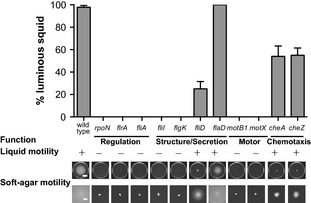
Entrance into a productive symbiosis with juvenile *Euprymna scolopes* by selected swimming-motility mutants. Squid were transiently exposed to the indicated strain for 24 h and the percentage that produced detectable luminescence at 48 h postcolonization was determined. Functional groups indicated beneath the strains correspond to those in Table 1. Liquid motility and soft-agar motility assays were performed as described in Experimental Procedures. White scale bars in wild-type soft-agar motility plates represent a distance of 20 mm in whole-plate views (top) and 5 mm in the higher magnification images (bottom).

To elucidate the roles of flagellar motility and chemotaxis in the initiation of the vibrio–squid symbiosis, strains representing the four primary functional groups of motility mutants isolated from the screen (Table 1) were exposed to juvenile *E. scolopes* to assay their ability to enter into a productive (i.e., detectably luminescent) symbiosis (Fig. 2). Regardless of their grouping, mutants that were unable to migrate through soft agar did not colonize juvenile squid to the level at which luminescence is detectable (i.e., ∼1% wild type [Ruby and Asato 1993]), even though all strains were able to produce wild-type levels of light in culture.

Transposon mutants in *fliD flaD cheA*, and *cheZ*, which were all able to migrate through soft agar to some level and also exhibited swimming motility in liquid, were able to colonize at least a portion of squid to luminous levels. The percentage of animals colonized by the *fliD*::Tn*erm* mutant, which has 19% of the soft-agar motility of wild type (Fig. 2), is similarly reduced relative to squid exposed to wild-type *V. fischeri*, suggesting that motility rate contributes to efficiency even under these permissive conditions, in which the squid are exposed to the inoculating bacteria for 24 h. The *cheA* and *cheZ* mutants colonized only about 50% of the squid to luminous levels, suggesting that, while chemotaxis is not essential for initiation, it is still required for wild-type colonization efficiency. As each mutant in a given functional class exhibited similar colonization ability, these experiments, even in the absence of genetic complementation, further clarify the roles of flagellar motility and chemotaxis in symbiotic initiation.

### FlrA regulates both flagellar and nonflagellar genes

To more fully examine the relationship between genes required for normal motility and those activated by FlrA, we coupled our functional studies with a complete transcriptional profile of the flagellar regulon in *V. fischeri*. Prokaryotic flagellar systems have been models for hierarchical regulation, and flagellar gene discovery based on transcriptional profiling has proven successful in other bacteria (Frye et al. 2006; Morris et al. 2008). To identify the flagellar regulon of *V. fischeri*, we used a whole-genome microarray analysis that compared the transcriptomes of the wild-type strain and an isogenic Δ*flrA*::*kan* strain. In the absence of FlrA, the expression of 131 genes was significantly reduced, including 39 predicted flagellar and chemotaxis genes (Table S3).

**Figure 3:**
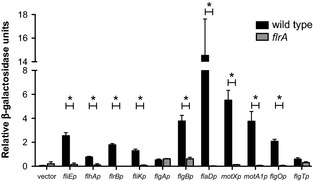
Flagellar-gene promoter activities in wild-type and *flrA*-mutant strains. Promoters for 11 genes were transcriptionally fused to *lacZ* as described in Experimental Procedures, and β-galactosidase activity was measured in wild type and the *flrA*::Tn*erm* mutant after growth in seawater-based tryptone (SWT) to an OD_600_ of ∼0.5. Note that not all promoters (e.g., *flgA*) are controlled by FlrA. Asterisks indicate both a significance difference at *P* ≤ 0.05 using a Student's *t*-test and a fold change ≥2.

Control of flagellar promoters by FlrA was confirmed using β-galactosidase transcriptional reporter fusions (Fig. 3). Of the nine flagellar promoters tested, eight constructs exhibited significantly reduced β-galactosidase activity in the *flrA*::Tn*erm* mutant relative to wild type. The *flgA'-lacZ*^*+*^ construct yielded similar levels of β-galactosidase activity regardless of strain background, consistent with both the microarray data (Table 1) and previous work performed with its ortholog in *V. cholerae* (Prouty et al. 2001). Of the novel genes required for normal flagellar elaboration, only the *flgO'-lacZ*^*+*^ reporter responded to the absence of FlrA, suggesting that the promoter serving *flgO* and *flgP* (discussed later), but not that controlling *flgT*, is activated by FlrA. Confirming the microarray data, the activity of the *flgT'-lacZ*^*+*^ reporter, while slightly reduced, was not significantly changed in the absence of FlrA.

**Figure 4:**
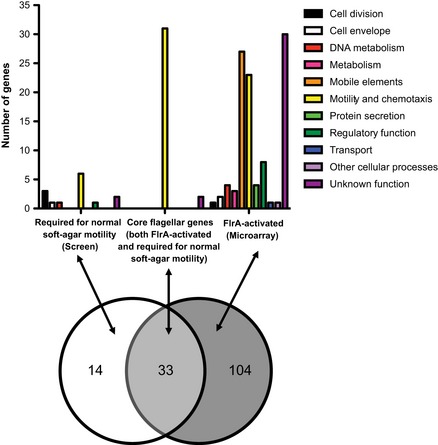
Comparison of soft-agar motility screening and microarray analyses. The set of genes required for normal soft-agar motility (genes disrupted in those mutants with severe defects; Table 1) was compared to the flagellar regulon (FlrA-activated genes; Table S3). The 33 genes present in both data sets are considered “core flagellar genes”, and include 31 predicted flagellar motility and chemotaxis genes, together with *flgO* and *flgP* (“unknown function”).

We compared (i) the genes in the microarray analysis that were activated by FlrA (i.e., downregulated in Δ*flrA*::*kan* relative to wild type) with (ii) the genes that the genetic screen suggested were required for motility. The overlap between these two groups was considered to constitute the “core flagellar genes” (Fig. 4). This core includes predicted flagellar and chemotaxis genes, as well as *flgO* and *flgP*. Some predicted flagellar genes, which we had anticipated finding in this core, were found in only one of the two data sets. For instance, *flaC*, which encodes one of the six flagellin proteins, is FlrA controlled but not required for flagellar motility (Millikan and Ruby 2004). Interestingly, a paralog of one flagellar gene, *fliL2*, (VF_2446), which can be found in many *Vibrio* species and whose role in motility is unknown, was reduced in the absence of FlrA. Conversely, the flagellum-specific ATP synthase-encoding gene *fliI* was required for soft-agar motility (Table 1), but was not significantly regulated in the FlrA transcriptomic study (Table S3). A small subset of predicted flagellar genes, including *flgA*, is absent from both data sets (data not shown), suggesting that either, (i) even in combination, these techniques are not fully comprehensive, or (ii) these genes are disassociated from the flagellar regulatory process in *V. fischeri*.

The FlrA-regulon also include a large number of genes predicted to be found among mobile elements, specifically the cryptic phages encoded on Chromosome 1 of *V. fischeri* ES114, as well as genes located on this strain's large conjugative plasmid. In addition, 17 genes were upregulated in the Δ*flrA*::*kan* strain between two- and fourfold, suggesting negative regulation by FlrA (Table S3); however, these targets were annotated to have functions unrelated to flagellar motility. As FlrA is not known to act directly to repress transcription, we postulate that regulation of these genes is indirect.

### Genes encoding proteins of unknown function are required for soft-agar motility and normal flagellar structure

**Figure 5:**
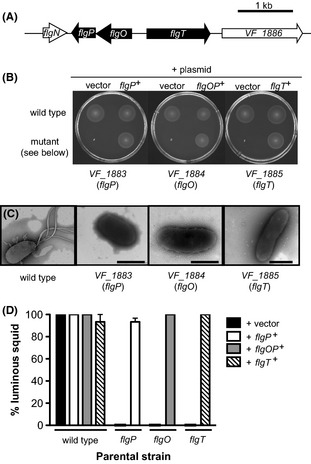
Mutants in the *Vibrio fischeri flgOP* and *flgT* loci. (A) Genomic organization of the *flgOP* and *flgT* loci. (B) Motility of indicated strains in seawater-based tryptone (SWT) containing 0.3% agar. (C) Negative-stained transmission electron micrographs of strains grown in SWT broth. Scale bars indicate 1 μm. (D) Complementation of *flgO flgP*, and *flgT* mutant colonization defects. Squid were transiently exposed to the indicated strain for 24 h, and a successful colonization was scored by the presence of detectable luminescence at 48 h postcolonization.

We further examined the unexpected mutants identified in the motility screen, beginning with four mutants disrupted in genes encoding hypothetical proteins. Three of these genes, *VF_1883* (*flgP*), *VF_1884* (*flgO*), and *VF_1885* (*flgT*), were described as associated with flagellar motility in *V. cholerae* recently (Cameron et al. 2008; Morris et al. 2008; Martinez et al. 2009), and we have adopted their nomenclature. The genomic organization of *flgO flgP*, and *flgT* suggests two transcriptional units: *flgOP* and *flgT*, which are located at one end of the flagellar locus on Chromosome 1 (Fig. 5A). In *V. fischeri*, all three mutants are completely amotile in soft agar (Fig. 5B). Further, while the *flgP*::Tn*erm* and *flgT*::Tn*erm* mutants can be complemented by expression on a plasmid, the *flgO*::Tn*erm* mutant cannot be complemented by expression of either *flgO*^*+*^ or *flgP*^*+*^ individually, and expression of both *flgO*^*+*^ and *flgP*^*+*^ is required to restore wild-type motility (Fig. 5B and data not shown), indicating at least part (but not all) of the defect in this mutant is due to polar effects on *flgP*. When examined by phase-contrast microscopy, these three mutants have normal cell morphology, but only the *flgP*::Tn*erm* mutant showed even an occasional motile cell (data not shown).

Because of the large defects in flagellar motility of these mutants, we used negative-staining TEM to determine whether the cells possessed flagella. Unlike wild-type *V. fischeri* cells, which present a tuft of sheathed, polar flagella, the vast majority of cells of the *flgO*::Tn*erm flgP*::Tn*erm*, and *flgT*::Tn*erm* mutants were aflagellate (Fig. 5C). However, flagellar-like structures could be observed in the media from all preparations and, at very low frequency, we observed what appeared to be a single, altered flagellum on *flgP*::Tn*erm* and *flgO*::Tn*erm* cells (data not shown). Therefore, *V. fischeri flgO flgP*, and *flgT* play an essential role in soft-agar motility, as well as in the normal presentation of flagella on the cell surface.

We also analyzed the ability of the mutants disrupted in *flgP flgO*, and *flgT* to enter into a symbiotic relationship with juvenile squid, and observed severe colonization defects by each of these mutants (Fig. 5D). Whereas none of the squid exposed to the mutant strains were colonized to luminous levels, the complemented strains were able to successfully enter into productive symbioses with juvenile squid. These data show that the *flgO*::Tn*erm flgP*::Tn*erm*, and *flgT*::Tn*erm* mutant strains are severely attenuated in colonization of juvenile squid, consistent with previous observations examining amotile mutants (Fig. 2, Millikan and Ruby 2003; Wolfe et al. 2004).

**Figure 6:**
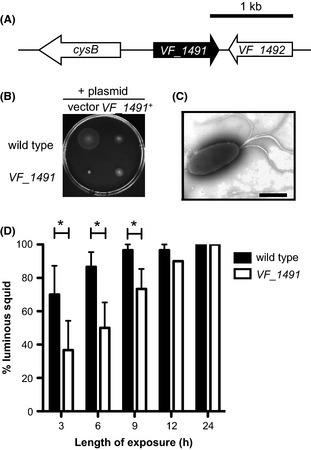
Motility and symbiotic-competence analysis of a *VF_1491* mutant. (A) Genomic organization of the *VF_1491* locus. (B) Motility of indicated strains in seawater-based tryptone (SWT) containing 0.3% agar. (C) Negative-stained transmission electron micrographs of the *VF_1491* mutant grown in SWT broth. Scale bars indicate 1 μm. (D) Relative effectiveness of *VF_1491* in colonizing juvenile squid. Squid were transiently exposed to either the *VF_1491* mutant or wild-type *Vibrio fischeri* for either 3, 6, 9, 12, or 24 h, and a successful colonization was scored by the presence of detectable luminescence at 48 h postcolonization. Asterisks indicate a significance difference at *P* ≤ 0.05 using a two-way repeated measure analysis of variance (ANOVA), with a post hoc Bonferroni correction.

The remaining mutant disrupted a gene of unknown function, *VF_1491*, predicted to be monocistronic (Fig. 6A) and found throughout the sequenced *Vibrio* species, but containing no recognizable SMART or PFAM domains (Letunic et al. 2012; Punta et al. 2012). The *VF_1491*::Tn*erm* mutant exhibited motility through soft agar at a rate that is only 9% that of wild type (Fig. 6B). When *VF_1491*^*+*^ is expressed *in trans* to complement the *VF_1491*::Tn*erm* strain, the soft-agar motility observed is greater than that of the *VF_1491*::Tn*erm* strain alone, but less than wild type carrying a vector control (Fig. 6B). Furthermore, expression of *VF_1491*^+^ *in trans* in the wild-type background leads to a reduction in soft-agar motility relative to wild type carrying the vector control. However, both mutant and wild type carrying plasmid-borne *VF_1491*^+^ swim at approximately the same level in the soft-agar motility assay (Fig. 6A). Because expression of *VF_1491* on a multicopy plasmid alters the motility of wild type, the stoichiometry of this protein may be important. Therefore, perhaps it is not surprising that the complementing construct does not fully restore wild-type motility to the *VF_1491*::Tn*erm* mutant. This phenotype is similar to what is seen with the chemotaxis protein CheW, in which both overexpression and disruption affect the chemotactic ability of *E. coli* (Sanders et al. 1989). Because soft-agar assays measure growth as well as motility (Wolfe and Berg 1989), we first confirmed that the *VF_1491*::Tn*erm* mutant grew comparably to wild-type cells (Fig. S2A). We next determined that the *VF_1491*::Tn*erm* cells are motile in liquid medium and present a tuft of polar flagella that is indistinguishable from that of wild-type cells (Fig. 6C). Supporting a potential role for VF_1491 in chemotaxis, examination of the *VF_1491*::Tn*erm* by phase-contrast microscopy revealed a strong tumbling bias (data not shown).

We examined the competence of the *VF_1491*::Tn*erm* mutant to enter into symbiosis with juvenile squid under permissive conditions (Fig. 6D). After 24 h of exposure to squid, both the *VF_1491*::Tn*erm* mutant and wild type colonized 100% of squid to luminous levels. However, at shorter exposure times, the *VF_1491*::Tn*erm* mutant colonized fewer squid to luminous levels than wild type, suggesting a reduced efficiency of symbiotic initiation in *VF_1491*::Tn*erm*-exposed squid.

### Mutations in predicted cell division genes lead to reduced soft-agar motility

**Figure 7:**
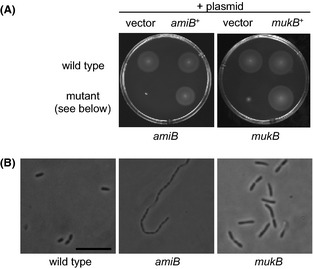
Soft-agar motility and phase-contrast microscopy of cell division mutants. (A) Motility of indicated strains in seawater-based tryptone (SWT) containing 0.3% agar. (B) Phase-contrast micrographs of SWT broth cultures of indicated strains. Scale bar indicate 5 μm.

Three mutants in genes predicted to play important roles during cell division were found to exhibit profound motility defects: *amiB mukF*, and *mukB* (Table 1). AmiB encodes an amidase involved in septal cleavage during cell division (Uehara et al. 2010). MukF and MukB, along with MukE, form a complex that functions as a bacterial condensin and mediates chromosome partitioning (Yamazoe et al. 1999; Case et al. 2004). The *amiB*::Tn*erm* mutant is unable to swim through soft agar, while the *mukF*::Tn*erm* and *mukB*::Tn*erm* mutants are each reduced to 22–23% of the wild-type motility level (Fig. 7A and Table 1). Because MukBEF form a complex we chose one, MukB, to pursue. To complement the *amiB*::Tn*erm* and *mukB*::Tn*erm* mutant phenotypes, we expressed the wild-type copy of each gene *in trans*, resulting in a restoration of normal motility (Fig. 7A). While the *amiB*::Tn*erm* and *mukB*::Tn*erm* mutants have no obvious growth defects (Fig. S2B), abnormal cell morphologies were observed when these mutants were examined by phase-contrast microscopy (Fig. 7B). Specifically, the *amiB*::Tn*erm* mutant forms long chains of cells, whereas the *mukB*::Tn*erm* mutant has increased numbers of doublets and triplets of cells. In both mutants, only the rare single cells and, even less frequently, doublets exhibit effective motility in liquid media, suggesting that the morphological defect is the primary reason for the mutants' reduced soft-agar motility. Because of these pleiotropic defects, we did not examine the ability of these strains to colonize juvenile squid.

### Paralogs have distinct phenotypes in soft-agar motility

**Figure 8:**
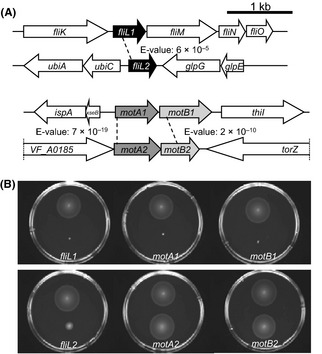
Genomic organization and soft-agar motility analysis of mutants in predicted paralogs of three flagellar genes (*fliL motA,* and *motB*). (A) Genomic organization of loci surrounding *fliL1 fliL2 motA1B1,* and *motA2B2*. *E*-values listed were determined by BLASTP analysis. (B) Motility of indicated strains in seawater-based tryptone (SWT) containing 0.3% agar. On all plates, the upper strain is wild-type *Vibrio fischeri*, and the lower strain carries a mutation in the gene indicated at the bottom of the plate.

The *V. fischeri* ES114 genome contains pairs of paralogs of the flagellar and chemotaxis genes *fliL motA*, and *motB* (Fig. 8A), as well as three *cheV* paralogs (Hussa et al. 2007). As our screen coverage was likely not saturating, we may have not obtained insertions in all of the paralogous loci. Therefore, when we observed transposon hits in only one paralog (e.g., *motA1*), we did not know whether the other paralog (*motA2*) was not hit in the screen, or whether mutation of the other paralog did not affect the cell's motility phenotype; thus, we chose to investigate these pairs directly.

We first investigated the two predicted paralogs of *motA* and *motB*, whose gene products are predicted to function in the flagellar motor apparatus. The presence of *motA2B2* in *V. fischeri* is unique among the completely sequenced *Vibrio* species currently in NCBI. MotA1 and MotB1 are more similar to the motor proteins of *V. cholerae*, PomA and PomB, than to *V. fischeri* MotA2 and MotB2. Additionally, transcription of *motA1B1*, but not *motA2B2,* is activated in the presence of FlrA (Table S3). To investigate the contributions of these four genes to normal flagellar motility, individual insertion mutations were constructed in *motA1 motB1 motA2*, and *motB2,* and their behaviors were observed. The mutants disrupted in either *motA1* or *motB1* were entirely defective in soft-agar motility, confirming the results we noted with the *motA1* and *motB1* transposon mutants (Fig. 8B and Table 1). In contrast, insertion mutations in *motA2* or *motB2* had no effect on soft-agar motility. Taken together, these data suggest that *motA1B1* encode the canonical flagellar motor proteins, and *motA2B2* are not required for flagellar motility, at least under the conditions assayed.

The presence of two paralogs encoding the predicted flagellar basal body–associated protein FliL is a conserved trait among the sequenced *Vibrio* spp (McCarter 2006). In *V. fischeri*, only *fliL1* is located in the flagellar locus and was found to be required for motility in our screen (Fig. 8A and Table 1). Unlike *motA1B1* and *motA2B2*, both *fliL1* and *fliL2* are activated by FlrA, and therefore, belong to the flagellar regulon of *V. fischeri* (Table S3). Specifically constructed insertion mutations in either *fliL1* or *fliL2* result in distinct defects in soft-agar motility (Fig. 8B). The *fliL1* mutant is amotile; however, because it is predicted to be part of a downstream operon, the insertion is likely to be polar on *fliMNOPQR* and, as such, the individual contribution of *fliL1* to motility cannot be clearly determined (Fig. 8A). In contrast, the motility of the monocistronic *fliL2* mutant is reduced relative to wild type, and can be complemented by *fliL2* expression *in trans* (Fig. S3). Thus, *fliL2* represents a previously unknown genetic determinant associated with flagellar motility in *V. fischeri*.

## Discussion

In this study, we (i) generated a 23,904 member transposon insertion library, a useful new tool for the study of *V. fischeri*, (ii) identified new genetic determinants of flagellar motility, (iii) utilized a *flrA* microarray as a discovery tool, and (iv) expanded on the importance of this behavior in the initiation of the squid–vibrio symbiosis.

### *Vibrio fischeri* arrayed mutant library

We describe the construction and validation of the *V. fischeri* ES114 MB mutant collection. By coupling the construction of this library with a phenotypic screen of a well-characterized behavior, rather than performing the screen after completion of the collection, we were able to assay quality throughout the library construction process, immediately address any problems, and verify that the strains in the collection are in good condition. For example, we observed highly variable soft-agar motility in a batch of ten 96-well trays prepared on the same day, and discarded them after determining they were likely damaged due to an unintended exposure to high temperature. This process also allowed us to obtain estimates of sibling membership in the library. These quality assurance measures establish the MB collection as a useful and immediately available tool for use by the squid–vibrio community. In fact, the collection has already been used to quickly locate mutants of interest by PCR screening of strain lysate pools (Studer et al. 2008), and to isolate a mutant with a cell surface phenotype (Post et al. 2012).

### Genetic determinants of soft-agar motility

Through genetic analysis, we identified mutants in 38 homologs of flagellar motility-associated genes required for normal soft-agar motility in *V. fischeri*. These data are largely consistent with studies into the genetic basis of motility, a behavior whose core machinery is well characterized, in other *Vibrio* spp (McCarter 1995; Cameron et al. 2008), and serve as confirmation of the depth of our screen. Surprisingly, we also isolated a mutant in *flaD*, one of the six flagellin-encoding genes in *V. fischeri*, which has been shown to be dispensable for normal motility in both *V. cholerae* (Klose and Mekalanos 1998a) and *V. anguillarum* (Milton et al. 1996). Our data, along with previous work (Millikan and Ruby 2004), indicate that the contributions of individual flagellin homologs vary between the multiple polar flagella of *V. fischeri* and the single polar flagellum of these other *Vibrio* spp. Future experiments with the flagellin mutants may provide insight into the long-standing question of the function(s) underlying the presence of multiple flagellins in the *Vibrionaceae* (e.g., does the *flaD* mutant produce fewer flagella per cell or similar numbers of shorter flagella?).

Our work has also identified several genetic determinants of motility in *V. fischeri* that have not been previously reported. First, we observed a strong association between normal cell morphology and soft-agar motility in *V. fischeri*. The *amiB* mutant formed chained cells that were amotile even in liquid; a homologous transposon mutant in *V. cholerae* exhibited reduced, but not entirely deficient, soft-agar motility (Rashid et al. 2003). Similarly, while mutants in the *muk* locus of *V. fischeri* were severely attenuated in soft-agar motility, this locus has not been shown to be associated with soft-agar motility in *V. cholerae*. While the basis for these differences between *V. fischeri* and *V. cholerae* are unknown, it is possible that a tighter regulation between flagellar motility and cellular division is important during the symbiotic lifestyle of *V. fischeri*. Other determinants of motility thus far unique to *V. fischeri* include VF_1491, a protein of unknown function, and FliL2, a paralog of the basal body protein FliL1. Neither of these genes has yet been identified in soft-agar motility screens of other *Vibrio* spp, despite the presence of homologs in many species of *Vibrio* and other gamma-proteobacteria.

Finally, whereas previous investigations of mutations in any one of the three paralogs of the chemotaxis protein CheV in *V. fischeri* identified no discernable reductions in soft-agar motility (Hussa et al. 2007), suggesting functional redundancy, we have shown that differences in soft-agar motility can be observed between mutants in the *fliL1* and *fliL2* genes, as well as in the *motA1B1* and *motA2B2* loci. In contrast to the dominant role of the *motA1B1* motor proteins in *V. fischeri* under the conditions of our assay, work in *Aeromonas hydrophila* has shown that the two pairs of *pomAB*-like genes identified in the genome have largely redundant functions (Wilhelms et al. 2009). Taken together, these results reinforce the idea that the roles of motility-gene paralogs cannot be predicted a priori and that, in some cases, they have likely diverged from an ancestral involvement in motility.

### Transcriptional profiling of the flagellar regulon

In the absence of the flagellar master activator, FlrA, expression levels of most flagellar and chemotaxis genes are reduced relative to wild type in both *V. fischeri*, as was seen with *V. cholerae* (Syed et al. 2009). One exception is the MCPs, which serve as receptors and recognize environmental signals. Whereas, in *E. coli* K12, all five of the MCPs are regulated by this species' master regulator, FlhDC (Zhao et al. 2007), in our transcriptional analysis only four of the 43 predicted MCPs were significantly regulated by FlrA. Similarly, only seven of the 44 MCPs in *V. cholerae* O395 were identified as part of its flagellar regulon (Syed et al. 2009). This difference may indicate a fundamental divergence in the way chemotaxis is regulated between the *Vibrionaceae* and *Enterobacteriaceae*. Beyond motility-gene expression, flagellar master regulators of a number of bacterial species both positively and negatively modulate genes that are not involved in flagellar motility. In *E. coli* K12, a predominant example is in the regulation of anaerobic metabolism (Pruss et al. 2003), while in *V. cholerae*, the flagellar regulon controls virulence gene expression, including the toxin coregulated pilin (Tcp) genes (Syed et al. 2009). Our data suggest that, while nonflagellar genes may be regulated by FlrA in *V. fischeri,* FlrA does not control either anaerobic metabolism or the *Vibrio*-specific Tcp genes, intimating that the flagellar regulation machinery has been coopted for differing functions even between closely related species of bacteria.

### Flagellar motility and chemotaxis in symbiosis

Flagellar motility and chemotaxis have been implicated, to different degrees, in many host–microbe interactions. However, both infection model and strain differences can impact the relative importance of these behaviors in *V. cholerae* pathogenesis, as was first observed under lab conditions by Richardson (1991). Similarly, flagellar and chemotaxis mutants of uropathogenic *E. coli* exhibit a range of phenotypes depending on the organ of interest and on whether the strains are assessed in single- or dual-strain (competitive) analyses (Lane et al. 2005; Wright et al. 2005). Even with these ambiguities, chemotaxis and flagellar motility have been proposed to play important roles in these, and many other, host–microbe associations (Rawls et al. 2007; Rolig et al. 2012).

In our screen, we identified *V. fischeri* mutants in 43 genes required for normal soft-agar motility, and assayed 14 of these strains for colonization of the juvenile squid light organ. In this more exhaustive study, we confirmed previous data (Graf et al. 1994; Millikan and Ruby 2003; Wolfe et al. 2004; Hussa et al. 2007) and showed that flagellar motility is required for entrance into a productive symbiosis. Interestingly, mutants disrupted in core chemotaxis genes were able to colonize juvenile squid, albeit with reduced success. These data, and those showing that mutants in *cheY* and *cheR* are significantly out-competed by wild type (Hussa et al. 2007; DeLoney-Marino and Visick 2012), suggest that chemotaxis is an important symbiotic behavior, especially under natural, competitive conditions, in which hatchling squid are likely exposed to a diversity of *V. fischeri* strains in the planktonic environment. To explain the different phenotypes for motility and chemotaxis mutants, we propose that either (i) nonchemotactic mutants are able to “blindly” locate the light organ, which would suggest that chemotaxis is required only over a short distance, and/or (ii) both nonchemotactic and chemotactic motility play a role in initiating colonization.

The specific stages at which chemotaxis and flagellar motility mediate colonization are not yet well characterized in any bacterium–animal interaction. The squid–vibrio system is posed as an excellent model to address both how and when these bacterial behaviors underlie symbiotic initiation, as colonization occurs naturally from the surrounding seawater and can be easily manipulated and observed. While they have been examined in plant symbioses (Gage et al. 1996), mechanisms of natural initiation are underrepresented in studies of animal–microbe interactions. As such, the complexity observed during initiation of the squid–vibrio symbiosis continues to illuminate general mechanisms that underlie host–microbe interactions and serves as a natural, yet experimentally tractable, model of mutualism.

## Supporting Information

Figure S1 Construction and soft-agar motility screening of a *Vibrio fischeri* transposon mutant collection. A schematic diagram demonstrating the method of library construction and motility screening utilized in this work.

Figure S2 Growth phenotypes of the *VF_1491* and cell division mutants. (A) Growth of wild-type *Vibrio fischeri* and the *VF_1491* mutant in SWT broth under microaerobic conditions. (B) Growth of wild-type *V. fischeri*, either the *mukB* mutant or the *amiB* mutant, in SWT broth under microaerobic conditions.

Figure S3 Complementation of the *fliL2* soft-agar motility defect. Motility of indicated strains in SWT medium containing 0.3% agar.

Table S1 Strains and plasmids used in this study.

Table S2 Primers used in this study.

Table S3 All genes differentially regulated in the absence of FlrA.
