# Supplementary material for: Genetic determinants of swimming motility in the squid light-organ symbiont Vibrio fischeri
Source: Microbiologyopen. 2013 Jun 12;2(4):576–94. doi: 10.1002/mbo3.96 (PMC3948606; doi:10.1002/mbo3.96)
Supplement: Table S1 — Strains and plasmids used in this study. [file mbo30002-0576-sd1.pdf]

Table S1. Strains and plasmids used in this study

Strain or Plasmid	Description	Reference or source
Strains		
V. fischeri		
MJM1100	ES114, sequenced wild-type light organ isolate	(Boettcher and Ruby, 1990)
DM159	Δ*flrA::kan*	(Millikan and Ruby, 2003)
CAB1625	*fliL1*::pCAB50 Campbell mutant	This work
CAB1626	*fliL2*::pCAB51 Campbell mutant	This work
CAB1627	*motA1*::pCAB52 Campbell mutant	This work
CAB1628	*motA2*::pCAB53 Campbell mutant	This work
CAB1629	*motB2*::pCAB54 Campbell mutant	This work
CAB1630	*motB1*::pCAB55 Campbell mutant	This work
MB06265	*flgP*::Tn*erm*	This work
MB06357	*motB1*::Tn*erm*	This work
MB06428	*cheY*::Tn*erm*	This work
MB08164	*flgI*::Tn*erm*	This work
MB08359	*VF_0171*::Tn*erm*	This work
MB08380	*rfbX*::Tn*erm*	This work
MB08627	*VF_2581*::Tn*erm*	This work
MB08701	*cheA*::Tn*erm*	This work
MB08705	*VF_0173*::Tn*erm*	This work
MB08726	ig*VF_0135*::Tn*erm*	This work
MB08840	*flhA*::Tn*erm*	This work
MB08886	*flrC*::Tn*erm*	This work
MB08888	*fliI*::Tn*erm*	This work
MB09672	*mutS*::Tn*erm*	This work
MB09956	*flgH*::Tn*erm*	This work
MB10022	23s rRNA::Tn*erm*	This work
MB11332	*VF_1697*::Tn*erm*	This work
MB12230	*fliM*::Tn*erm*	This work
MB12341	*flgK*::Tn*erm*	This work
MB12561	*motX*::Tn*erm*	This work
MB13452	*rffH*::Tn*erm*	This work
MB14051	*VF_0172*::Tn*erm*	This work
MB14478	*flgT*::Tn*erm*	This work
MB14580	*flgE*::Tn*erm*	This work
MB14746	*rmlB*::Tn*erm*	This work
MB15443	*flhG*::Tn*erm*	This work
MB15490	*VF_0192*::Tn*erm*	This work
MB15821	*fliA*::Tn*erm*	This work
MB16040	23s rRNA::Tn*erm*	This work
MB16329	*flgL*::Tn*erm*	This work
MB16504	*flaA*::Tn*erm*	This work
MB16653	*VF_0077*::Tn*erm*	This work
MB18981	*fliG*::Tn*erm*	This work
MB19388	*flhF*::Tn*erm*	This work
MB19520	*fliL1*::Tn*erm*	This work
MB19624	*cheW*::Tn*erm*	This work
MB20044	*VF_A0058*::Tn*erm*	This work
MB20794	*fliN*::Tn*erm*	This work
MB20979	*cheZ*::Tn*erm*	This work
MB21386	*flgO*::Tn*erm*	This work
MB21407	*flrA*::Tn*erm*	This work

MB21447	*fliR*::Tn*erm*	This work
MB21566	*flgN*::Tn*erm*	This work
MB22953	*fliF*::Tn*erm*	This work
MB23025	*flgF*::Tn*erm*	This work
MB23130	*mshB*::Tn*erm*	This work
MB23533	*flgG*::Tn*erm*	This work
MB23837	*cheB::*Tn*erm*	This work
MB24277	*VF_1491*::Tn*erm*	This work
MB24439	*flaD*::Tn*erm*	This work
MB24714	*flrB*::Tn*erm*	This work
MB25656	16s rRNA::Tn*erm*	This work
MB26712	*motY*::Tn*erm*	This work
MB26857	16s rRNA::Tn*erm*	This work
MB27080	*fliD*::Tn*erm*	This work
MB28068	ig*VF_1874*::Tn*erm*	This work
MB28468	*flgD*::Tn*erm*	This work
MB28617	23s rRNA::Tn*erm*	This work
MB28641	*motA1*::Tn*erm*	This work
MB29802	*fliK*::Tn*erm*	This work
MB30445	*rpoN*::Tn*erm*	This work
MB30578	*fliH*::Tn*erm*	This work
MB31054	ig*VF_1837*::Tn*erm*	This work
MB31274	*mukF*::Tn*erm*	This work
MB32667	*can*::Tn*erm*	This work
MB32946	*amiB*::Tn*erm*	This work
MB33180	16s rRNA::Tn*erm*	This work
MB33191	*VF_0189*::Tn*erm*	This work
MB33314	*mukB*::Tn*erm*	This work
MB33650	*VF_0174*::Tn*erm*	This work
E. coli		
DH5*α*-*λ*pir	Cloning strain	(Hanahan, 1983)
Plasmids		
pMJM10	Plasmid used for Tn*erm* transposition, kan^{R}	This work
pEVS122	*oriR6K*-based suicide vector, erm^{R}	(Dunn *et al*., 2005)
pVSV105	pES213-based plasmid used for complementation, cam^{R}	(Dunn *et al*., 2006)
pAKD701	pES213-based plasmid used for reporter fusions, kan^{R}	(Dunn and Stabb, 2008)
pEVS104	conjugative helper plasmid, kan^{R}	(Stabb and Ruby, 2002)
pCAB52	pEVS122 containing a fragment of *motA1* ORF	This work
pCAB55	pEVS122 containing a fragment of *motB1* ORF	This work
pCAB53	pEVS122 containing a fragment of *motA2* ORF	This work
pCAB54	pEVS122 containing a fragment of *motB2* ORF	This work
pCAB50	pEVS122 containing a fragment of *fliL1* ORF	This work
pCAB51	pEVS122 containing a fragment of *fliL2* ORF	This work
pCAB11	pVSV105 with the *flgOP* complementing fragment	This work
pCAB12	pVSV105 with the *flgP* complementing fragment	This work
pCAB60	pVSV105 with the *flgT* complementing fragment	This work
pCAB58	pVSV105 with the *VF_1491* complementing fragment	This work
pCAB59	pVSV105 with the *amiB* complementing fragment	This work

pCAB66	pVSV105 with the *mukB* complementing fragment	This work
pCAB65	pVSV105 with the *mutS* complementing fragment	This work
pCAB56	pVSV105 with the *fliL2* complementing fragment	This work
pCAB36	pAKD701 with the *fliE* promoter	This work
pCAB37	pAKD701 with the *flhA* promoter	This work
pCAB38	pAKD701 with the *flrB* promoter	This work
pCAB39	pAKD701 with the *fliK* promoter	This work
pCAB47	pAKD701 with the *flgA* promoter	This work
pCAB40	pAKD701 with the *flgB* promoter	This work
pCAB44	pAKD701 with the *flaD* promoter	This work
pCAB41	pAKD701 with the *motX* promoter	This work
pCAB46	pAKD701 with the *motA1* promoter	This work
pCAB48	pAKD701 with the *flgO* promoter	This work
pCAB49	pAKD701 with the *flgT* promoter	This work
