# Supplementary material for: Genetic determinants of swimming motility in the squid light-organ symbiont Vibrio fischeri
Source: Microbiologyopen. 2013 Jun 12;2(4):576–94. doi: 10.1002/mbo3.96 (PMC3948606; doi:10.1002/mbo3.96)
Supplement: Table S2 — Primers used in this study. [file mbo30002-0576-sd2.pdf]

Table S2. Primers used in this study

Primer name	Sequence (5'-3')
T7US-F2	CTAGATAATACGACTCACTATAGGGCGGCC
T7US-R2	GCCCTATAGTGAGTCGTATTAT
MseTspUS-F	GAGACAGGTCGACCTGCAGGGTTAATTGGGAAAGCCACGTTGTGTCTC
MseTspUS-R	GAGACACAACGTGGCTTTCCCAATTAACCCTGCAGGTCGACCTGTCTC
T7DS-pcrF2	GAGGATGTCGGTACCCCCTATAGTGAGTCGTATTAGTACAACTGAG
T7DS-pcrR	GTGACGTCAGGTACCAGATGTGTATAAGAGACAGAATTAATCCGTAGCGTCC TGAACGG
170Int2	AGCTTGCTCAATCAATCACC
ARB1	GGCCACGCGTCGACTAGTACNNNNNNNNNNGATAT
170Ext3	GCAGTTCAACCTGTTGATAGTACG
ARB2	GGCCACGCGTCGACTAGTAC
170Int3	CAAAGCAATTTTGAGTGACACAGG
170Seq1	AACACTTAACGGCTGACA
fliL1_campbellF	GCCCCGGGTGCCGCTTTAGAAGCGAATATGCC
fliL1_campbellR	GCGCATGCTTCGGCCACGTGGTTCTCTTAACT
fliL2_campbellF	GCCCCGGGAGAAGTCGCACCTCAAATGGGCTA
fliL2_campbellR	GCGCATGCTCTTATTTCTTCACGACCCGCCAG
motA1_campbellF	GCCCCGGGCAATGGTACTGGCTGGTGGTATA
motA1_campbellR	GCGCATGCCTTTACGAGCCGCATCAGCCATTT
motB1_campbellF	GCCCCGGGATTGATGGAGAATCGGATCGTGCTG
motB1_campbellR	GCGCATGCCACTAATCTCGCGCTCTAGTGCTT
motA2_campbellF	GCCCCGGGCGGCTTATTGATGGCGTTTGGTGT
motA2_campbellR	GCGCATGCTCGTCACGTTTGTCTCGTCAAGCA
motB2_campbellF	GCCCCGGGAAAGTGGTTGATACGTCGGCTCAC
motB2_campbellR	GCGCATGCCGACCTCTTTCATCGCTTCTGAA
flgO_compF	GCTCTAGAACTTAAGATCCTTTAGGCGGC
flgP_compR	GCTCTAGACTAACACACAGGAAACAGCTATGAAACATTGGTTTTTATTA
flgP_compF	GCGGTACCACCGAGGGCAATAGCATTATGTCG
flgT_compF	GCTCTAGATGGCAATTGGTGAACGAGCA
flgT_compR	GCGGTACCAACAACACGCTAGGCACTGT
1491_compF	GCTCTAGAGCAGTCGATGATACATTCAAACTGTG
1491_compR	GCGAGCTCTCCACAGTGGTGTTCCTGTT
amiB_compF	GCTCTAGACTAACACACAGGAAACAGCTGTGTTAATCGGTAAATATTTTCGA
amiB_compR	GCGGTACCTTGGTTGGCTAATCGTGCTGGT
mukB_compF	CCTAACACACAGGAAACAGCTATGATTGAACGTGGTAAATA
mukB_compR	ACGCATTCCGCTTCTGTTGT
mutS_compF	AACTTCTTCACTAACCGCGCCA
mutS_compR	TGGCTGATTCATGGCTGCAT
fliL2_compF	GCTCTAGAACCCACGATTGCTGATCACCTT
fliL2_compR	GCGGTACCACAGCGCATTTAGCAGGCTT
fliE_fusionF	GCCCTAGGACCAGAGCAACAAGTACCGT
fliE_fusionR	GCGTCGACTTAGGCTTTCGCAAACATTGCA
flhA_fusionF	GCCCTAGGGACGCGACTACCAATATCCA
flhA_fusionR	GCGTCGACTTACGGTATGGGTAAAATTACCA
flrB_fusionF	GCCCTAGGTCGAATGATTTACCAGAAGA
flrB_fusionR	GCGTCGACTTACTTGTGTATCAAGTAAAATAACA

fliK_fusionF	GCCCTAGGTCACAAGTGGATAATTGCCA
fliK_fusionR	GCGTCGACTTACTCTTTTAGCTCTTCAATAAAGCCT
flgA_fusionF	GCCCTAGGAGAAACCTTGAATTGTACGGT
flgA_fusionR	GCGTCGACTTAGAAACTTATAAATAGGCCGA
flgB_fusionF	GCCCTAGGTGAAATCTCAAGTGTTAAATCA
flgB_fusionR	GCGTCGACTTATGCTTTATAGCGAGGAGT
flaD_fusionF	GCCCTAGGACTATGCCAAAGCCGGGGCT
flaD_fusionR	GCGTCGACTTAACTGTTAATTTTAAGACCTGA
motX_fusionF	GCCCTAGGTGAAGATCTAACAGGCGTGCCA
motX_fusionR	GCGTCGACTTAAAACAGTCGAAATAATTGGT
motA1_fusionF	GCCCTAGGTTGATTGACGACAGAATCCA
motA1_fusionR	GCGTCGACTTAACCAACTACGATTAAAACCGA
flgO_fusionF	GCCCTAGGTATCGGCCCCAGAAAACAGCA
flgO_fusionR	GCGTCGACTTAGCGTGGGGTATCAGCCAGCA
flgT_fusionF	GCCCTAGGGTCGTATTCGATGTTACAAACTGTTCA
flgT_fusionR	GCGTCGACTTATGCCGCATCTTTACTGGTTAAGA
