# Supplementary material for: Genetic determinants of swimming motility in the squid light-organ symbiont Vibrio fischeri
Source: Microbiologyopen. 2013 Jun 12;2(4):576–94. doi: 10.1002/mbo3.96 (PMC3948606; doi:10.1002/mbo3.96)
Supplement: Table S3 — All genes differentially regulated in the absence of FlrA. [file mbo30002-0576-sd3.pdf]

Table S3. All genes differentially regulated in the absence of FlrA

Locus Tag	Fold change	Gene	Description
VF_0094	5.7		Diguanylate cyclase
VF_0714	6.3	*motA1*	Flagellar motor protein
VF_0715	3.4	*motB1*	Flagellar motor protein
VF_0777	10.2		Methyl-accepting chemotaxis protein
VF_0915	4.5		Hypothetical protein
VF_0920	49.5		Hypothetical protein
VF_0926	2.6	*motY*	Flagellar motor protein
VF_1125	2.5		Hypothetical protein
VF_1330	2.5		Carbohydrate binding domain-containing protein
VF_1422	2.2		von Willebrand factor type A domain protein
VF_1427	2.2	*aslA*	Acrylsulfatase like protein
VF_1493	2.6		Hypothetical protein
VF_1556	3.4	*pldA*	Outer membrane phospholipase A
VF_1559	2.4		Hypothetical protein
VF_1771	2.2	*prkA*	Serine kinase
VF_1774	2.3	*ycgB*	SpoVR family protein
VF_1789	17.4		Methyl-accepting chemotaxis protein
VF_1827	2.6	*cheW*	Chemotaxis coupling protein
VF_1829	2.3	*parA2*	Chromosome partioning protein
VF_1830	2.1	*cheB*	Chemotaxis methyl esterase
VF_1831	2.0	*cheA*	Chemotaxis histidine kinase
VF_1832	2.2	*cheZ*	Chemotaxis phosphatase
VF_1833	2.2	*cheY*	Chemotaxis response regulator
VF_1836	2.6	*flhF*	Flagellar regulator
VF_1839	2.3	*flhB*	Flagellar biosynthesis protein
VF_1840	2.9	*fliR*	Flagellar biosynthesis protein
VF_1841	3.9	*fliQ*	Flagellar biosynthesis protein
VF_1842	3.2	*fliP*	Flagellar biosynthesis protein
VF_1843	7.1	*fliO*	Flagellar biosynthesis protein
VF_1844	6.4	*fliN*	Flagellar motor switch component
VF_1845	6.8	*fliM*	Flagellar motor switch protein
VF_1846	8.1	*fliL1*	Flagellar basal body-associated protein
VF_1847	9.2	*fliK*	Flagellar hook length control protein
VF_1851	6.7	*fliG*	Flagellar motor switch protein
VF_1852	6.6	*fliF*	Flagellar M-ring protein
VF_1853	16.1	*fliE*	Flagellar hook-basal body protein
VF_1854	8.1	*flrC*	Flagellar two-component response regulator
VF_1855	16.1	*flrB*	Flagellar two-component sensor kinase
VF_1856	17.5	*flrA*	Flagellar igma-54-dependent transcriptional activator
VF_1858	8.8	*fliS*	Flagellin chaperone
VF_1859	8.4	*flaI*	Flagellar protein
VF_1860	7.4	*fliD*	Flagellar hook-associated protein 2
VF_1861	22.7	*flaG*	Flagellar protein
VF_1862	43.3	*flaE*	Flagellin
VF_1863	51.5	*flaD*	Flagellin
VF_1864	19.7	*flaC*	Flagellin
VF_1865	5.1	*flaB*	Flagellin
VF_1866	32.0	*flaA*	Flagellin
VF_1867	8.7	*flgL*	Flagellar hook-associated protein 3
VF_1868	41.3	*flgK*	Flagellar hook-associated protein 1
VF_1869	12.7	*flgJ*	Flagellum-specific peptidoglycan hydrolase

VF_1870	3.5	*flgI*	Flagellar P-ring protein
VF_1871	6.3	*flgH*	Flagellar L ring protein
VF_1872	12.3	*flgG*	Flagellar distal rod protein
VF_1873	9.6	*flgF*	Flagellar proximal rod protein
VF_1874	5.7	*flgE*	Flagellar hook protein
VF_1875	7.5	*flgD*	Flagellar hook capping protein
VF_1876	8.5	*flgC*	Flagellar proximal rod protein
VF_1877	8.2	*flgB*	Flagellar proximal rod protein
VF_1882	2.5	*flgN*	Chaperone
VF_1883	8.7	*flgP*	Flagellar motility-associate protein
VF_1884	10.1	*flgO*	Flagellar motility-associate protein
VF_2009	3.3		Hypothetical protein
VF_2010	2.4		Hypothetical protein
VF_2011	2.2		Hypothetical protein
VF_2012	2.3		Major capsid protein
VF_2013	3.3		Probable capsid scaffolding protein
VF_2015	2.9		Capsid scaffolding protein
VF_2016	2.5		Hypothetical protein
VF_2017	2.9		L-alanyl-D-glutamate peptidase
VF_2025	2.9		Phade baseplate assembly protein
VF_2026	2.6		Phage tail protein
VF_2027	3.1		Hypothetical protein
VF_2029	2.0		Hypothetical protein
VF_2031	2.4		Conserved hypothetical protein
VF_2033	3.2		Hypothetical bacteriophage protein
VF_2034	3.2		Hypothetical protein
VF_2035	2.5		Hypothetical protein
VF_2036	3.4		Phage regulatory protein (CII)
VF_2079	12.1	*flaF*	Flagellin
VF_2317	11.1	*motX*	Flagellar motor protein
VF_2446	2.2	*fliL2*	Flagellar basal body-associated protein
VF_A0071	2.3	*hnoX*	Heme nitric oxide binding protein
VF_A0287	16.1		Hypothetical protein
VF_A0325	5.9		Methyl-accepting chemotaxis protein
VF_A0502	6.3		Hypothetical protein
VF_A0564	11.2	*traF*	TraF protein
VF_A0677	10.3		Methyl-accepting chemotaxis protein
VF_A0715	7.5		Chitodextrinase precursor
VF_A0788	2.7		Hypothetical protein
VF_A0802	2.7	*cheV2*	CheW/CheY hybrid
VF_A0834	9.7		NirV precursor
VF_A0856	6.7		Hypothetical protein
VF_A1014	4.2		Diguanylate phosphodiesterase
VF_A1015	4.3	*rpoQ*	Quorum-sensing regulated RpoS-like sigma factor
VF_A1016	2.4		Two-component sensory histidine kinase
VF_A1017	2.7		Two-component response regulator
VF_B20	3.5		Hypothetical protein
VF_B21	4.8		Hypothetical protein
VF_B22	3.0		Hypothetical protein
VF_B23	3.6		Hypothetical protein
VF_B24	3.8		Hypothetical protein
VF_B25	3.4		Hypothetical protein
VF_B26	3.1		Hypothetical protein
VF_B27	3.9		Hypothetical protein
VF_B28	3.6		Hypothetical protein
VF_B29	3.4	*sbcC*	Exonuclease

VF_B30	3.1		Hypothetical protein
VF_B31	3.4		Hypothetical protein
VF_B32	3.3		Transporter
VF_B33	3.0		Hypothetical protein
VF_B34	2.9		Hypothetical protein
VF_B37	2.9		Hypothetical protein
VF_B38	3.4		Attachment mediating VirB2-like protein
VF_B39	2.7		VirB4 ATPase
VF_B40	4.0		Channel protein VirB8
VF_B41	3.0		Channel protein VirB9
VF_B42	3.2		Channel protein VirB10
VF_B43	2.6		VirB11 ATPase
VF_B44	2.8		Protein VirD4
VF_B45	3.1		Single-strand DNA binding protein
VF_B46	4.3		DNA topoisomerase III
VF_B47	3.6		Outer membrane protein
VF_B48	4.1		Hypothetical protein
VF_B49	3.2		Hypothetical protein
VF_B50	2.7		Relaxase
VF_B51	2.8		Hypothetical protein
VF_B52	2.8		Hypothetical protein
VF_B53	3.0		Hypothetical protein
VF_B54	3.3		Attachment mediating VirB5-like protein
VF_B55	2.5		Channel protein VirB6
VF_0349	-2.2		Chain length regulator, EPS biosynthesis protein
VF_0350	-2.2		Polysaccharise export periplasmic protein
VF_0352	-3.0		UDP-glucose lipid carrier transferase
VF_0510	-3.7		Fimbrial protein
VF_A0191	-2.4		FhuE receptor precursor
VF_A0193	-2.0		TolQ protein
VF_A0199	-2.1	*oppD*	Chitooligosaccharide transport ATP-binding protein
VF_A0328	-2.1	*yiaD*	Outer membrane lipoprotein
VF_A0329	-3.4		Hypothetical protein
VF_A0330	-3.0		Hypothetical protein
VF_A0331	-2.6		Hypothetical protein
VF_A0332	-2.7		TonB system receptor
VF_A0333	-2.5	*ptrB*	Oligopeptidase B
VF_A0610	-2.5		Hypothetical protein
VF_A0848	-2.4		Hypothetical protein
VF_A0885	-2.3		Hypothetical protein
VF_A1038	-2.1		Diguanylate phosphodiesterase

[a] Fold change of wild-type expression relative to Δ*flrA* condition, (*i.e.*, positive values indicate putative activation by FlrA)
